# Treatment-emergent mutations in myelodysplastic syndrome with del(5q) – lenalidomide related or disease-intrinsic clonal evolution?

**DOI:** 10.1038/s41408-024-01027-5

**Published:** 2024-03-18

**Authors:** Mostafa Abdallah, Kaaren Reichard, Naseema Gangat, Ayalew Tefferi

**Affiliations:** Mayo Clinic, Rochester, MN USA

**Keywords:** Myelodysplastic syndrome, Cancer genetics

**TO THE EDITOR:**

Myelodysplastic syndrome (MDS) with del(5q) is a subcategory of MDS without excess blasts (<5% in bone marrow and 2% peripheral blood) and is characterized by the presence of an often isolated del5q cytogenetic abnormality. In addition, the diagnosis according to the International Consensus Classification (ICC) allows for the presence of one additional cytogenetic abnormality, exclusive of -7/del(7q), while requiring the absence of “multi-hit” *TP53* mutation [1]. Patients with MDS-del(5q) also harbor other somatic mutations, with the two most frequent being *TP53* (~20% incidence at diagnosis) and *SF3B1* (~18%) [2]. These two mutations display significant clustering and are more prevalent in leukemic phase disease (~50% incidence), suggesting pathogenetic contribution to leukemic progression [2]. Furthermore, *TP53* variant allele frequency (VAF) > 22% has been shown to portend inferior overall and leukemia-free survival [2] and, along with therapy-related disease, was recently identified as the most prominent risk factor for survival in MDS-del(5q) [3]. The current standard of care in MDS-del(5q) is lenalidomide monotherapy for the alleviation of anemia [4, 5]. The emergence of *TP53* mutations during lenalidomide therapy of patients with MDS-del(5q) has previously been published [6–8]. In the current report, we extend these observations in an unselected cohort of 10 Mayo Clinic patients with MDS-del(5q) in whom next-generation sequencing (NGS) information was available before and after treatment with lenalidomide. The study was conducted under institutional review board-approved minimum risk protocols that allowed retrospective collection and analysis of patient history. Diagnosis of MDS-del(5q) and therapy-related qualification was according to ICC criteria [1]. Patient selection was based on the availability of NGS-derived mutation information before starting treatment with lenalidomide and at least one additional NGS analysis during treatment with lenalidomide.

Table 1 illustrates the mutational landscape in each of the 10 study patients (median age 75 years, range 65–79; 6 males), both before the start of treatment (1st NGS) with lenalidomide and a second-time point (2nd NGS) during treatment with lenalidomide; therapy-related disease was documented in 2 patients (patients 7 and 8). Baseline (1st) NGS study was performed before initiation of treatment with lenalidomide in all but one patient who was studied within 6 weeks of treatment with lenalidomide (Table 1; patient 2). The median time from initial diagnosis of MDS-del(5q) to 1st NGS study was 16 months (range 0–80). Mutation profiling at baseline (1st NGS; prior to lenalidomide therapy) disclosed one (10%) patient with monoallelic *TP53* mutation (VAF 19%; patient 4; Table 1) and two other patients (patients 1 and 2) with *SF3B1* mutation (VAF 8% and 14%); one of the latter *SF3B1*-mutated patients (patient 2) also harbored an *ASXL1* mutation (VAF 37%). The second NGS study during lenalidomide therapy was performed in all 10 patients at a median of 27 months (range 10-58) from the time of the first NGS study (Table 1). New *TP53* mutations emerged in 3 (33%) of 9 patients in whom the mutation was not detected at baseline (patients 1, 2, and 3; Table 1); the mutations were monoallelic (VAF 31%) in patient 1 (19 months from the 1st NGS study) and biallelic in the other two, with VAF of 22%/12% (patient 2; 49 months from the 1st NGS study) in one and 9%/3% (patient 3; 58 months from the 1st NGS study) in the other. In one patient (patient 4) harboring a *TP53* mutation at baseline (1st NGS study), *TP53* VAF decreased from 19% to 6% at 13-month interval (Table 1). Among the 3 patients with treatment-emergent *TP53* mutations, two were known to harbor *SF3B1* mutations at baseline (patient 1 with VAF 14% and patient 2 with VAF 8%) with patient 2 losing the *SF3B1* mutation at 49 months interval while *SF3B1* VAF increased in patient 1 from 14% to 32% at 19 months interval. Another patient (patient 9) acquired a new *SF3B1* mutation (VAF 5%), during the 2nd NGS study at 25 months interval (Table 1); the latter patient also acquired *CEBPA* (VAF 26%) and *WT1* (VAF 2%) mutations at the same time (Table 1). Additional lenalidomide treatment-emergent mutations included *ASXL1* (VAF 4%) and *IDH1* (VAF 4%) in patient 3 at 58 months interval and *RUNX1* (VAF 2%) in patient 1 at 19 months interval; all three patients were those with treatment-emergent *TP53* mutations (Table 1).

**Table 1:** Next-generation sequencing results in 10 patients with myelodysplastic syndrome with del(5q) before and after treatment with lenalidomide^a^.

Lenalidomide treatment start date		TP53-VAF		*SF3B1*-VAF		*ASXL1*-VAF		*IDH1*-VAF		*RUNX1*-VAF		*CEBPA*-VAF		*WT1*-VAF	
	Pts	1st NGS (date)	2nd NGS (date) Post-Rx time	1st NGS	2nd NGS	1st NGS	2nd NGS	1st NGS	2nd NGS	1st NGS	2nd NGS 3rd NGS	1st NGS	2nd NGS	1st NGS	2nd NGS
7/12/2021	1	No (5/4/2021)	Yes-31% (12/15/2022) *19 months*	Yes-14%	Yes-32%	No	No	No	No	No	No Yes-2%	No	No	No	No
10/29/2017	2	No (12/19/2017)	Yes-22%/12% (1/18/2022) *49 months*	Yes-8%	No	Yes-37%	Yes-44%	No	No	No	No	No	No	No	No
10/3/2018	3	No (6/8/2018)	Yes-9%/3% (4/7/2023) *58 months*	No	No	No	Yes-4%	No	Yes-4%	No	No	No	No	No	No
5/5/2022	4	Yes-19% (5/5/2022)	Yes-6% (6/2/2023) *13 months*	No	No	No	No	No	No	No	No	No	No	No	No
2/16/2017	5	No (2/2/2017)	No (1/25/2021) 47 months	No	No	No	No	No	No	No	No	No	No	No	No
11/03/2020	6	No (5/30/2019)	No (9/3/2021) 28 months	No	No	No	No	No	No	No	No	No	No	No	No
11/12/2020	7	No (9/30/2020)	No (7/26/2022) *22 months*	No	No	No	No	No	No	No	No	No	No	No	No
12/1/2020	8	No (10/20/2020)	No (11/12/2022) *25 months*	No	Yes-5%	No	No	No	No	No	No	No	Yes -26%	No	Yes-2%
11/2/2019	9	No (9/4/2018)	No (10/14/2021) *37 months*	No	No	No	No	No	No	No	No	No	No	No	No
10/9/2018	10	No (10/9/2018)	No (8/6/2019) *10 months*	No	No	No	No	No	No	No	No	No	No	No	No

^a^In all but one patient, baseline NGS was performed prior to initiation of treatment with lenalidomide while in the remaining one patient (patient #2) baseline NGS was performed in the first 6 weeks of treatment with lenalidomide.

Cytogenetic information at the time of diagnosis showed sole del(5q) in 8 patients and one other additional cytogenetic abnormality in 2 patients; patient 3 with del(17)(q25) in 14 of 15 metaphases and patient 10 with del(12)(p11.2p13) in 8 of 11 metaphases. Cytogenetic information at the time of the 2nd NGS study was also available in 9 patients and revealed no changes in 7 cases and disapperance of del(5q) in 2 patients (patients 2 and 8); both patients with lenalidomide treatment-associated disapperance of del(5q) started with only two abnormal metaphases at baseline. Transfusion-dependent anemia was documented in 1 patient at the time of the 1st NGS study (patient 1) and remained transfusion-dependent at the time of the 2nd NGS study but showed a steep decline in platelet count (174 to 53 × 10(9)/L; Table 1); the particular patient had responded to lenalidomide therapy and had become transfusion-independent in-between the 1st and 2nd NGS studies but had lost this response at the time of the 2nd NGS study, concurrent with the emergence of a new *TP53* mutation (VAF 31%; Table 1). Of note, patient 1 was recently described in a case report where salvage therapy with attenuated dose decitabine was shown to be successful in eradicating the *TP53* mutation and resumption of a transfusion-independent state [8]. Hemoglobin levels in the other 2 patients with treatment-emergent *TP53* mutations either improved (patient 2) or worsened (patient 3); the latter patient had also acquired new *ASXL1* (VAF 4%) and *IDH1* (VAF 4%) mutations (Table 1). Hemoglobin levels in four of the remaining 7 patients showed a decline, including patient 8 who acquired new *SF3B1* (5%), *CEBPA* (26%), and *WT1* (VAF 2%) mutations (Tables 1 and 2) or either an improvement or no change in 3 cases (Table 2). At last follow-up, all three patients with treatment-emergent *TP53* mutations were alive at 10 (patient 1), 52 (patient 2), and 44 (patient 3) months post-lenalidomide therapy; of note, the *TP53* mutations in the latter two patients were biallelic. None of the three patients with treatment-emergent *TP53* mutations were previously exposed to chemotherapy or radiotherapy and none experienced leukemic transformation; the latter was documented in patients 6 and 10, neither of whom displayed any mutation at baseline or during treatment with lenalidomide (Table 1).

**Table 2:** Complete blood count values for 10 patients with myelodysplastic syndromes with del(5q) at the time of first (prior to treatment with lenalidomide) and second (during treatment with lenalidomide) NGS studies.

pt		Hemoglobin g/dL (or Tx-dep)			Platelets x 10(9)/L			Leukocytes x 10(9)/L	
	At Dx	1st NGS	2nd NGS	At Dx	1st NGS	2nd NGS	At Dx	1st NGS	2nd NGS
1	8.9	Tx-dep	Tx-dep	152	174	53	4.2	6.2	3.2
2	9.6	9.6	11.7	142	142	149	5.3	5.3	17.4
3	8.4	8.4	Tx-dep	584	584	215	3.7	3.7	3.6
4		10.1	15.7		232	143		5.6	5.3
5	9.6	9.9	9.6	277	303	269	4	3.9	4.12
6	11.9	9.9	8.4	451	729	101	6	6.6	4.3
7	14.2	14.2	14.5	831	831	629	8.8	5.68	4.29
8	12.2	10.7	7.3	102	90	86	3.1	1.7	2
9	8.3	8.3	Tx-dep	132	132	123	3.5	3.5	3.9
10	10.2	10.2	8	198	395	88	6.3	2.78	1.01

*NGS* next generation sequencing, *Tx-dep* red cell transfusion-dependent, *Dx* diagnosis; *1st NGS*, prior to treatment with lenalidomide, *2nd NGS* during treatment with lenalidomide, *pt* patient

The current report confirms previously published observations regarding treatment-emergent *TP53* mutations in patients with MDS-del(5q) receiving lenalidomide therapy [9–11]. In one of these reports, Lode et al., described 24 patients with MDS-del(5q) who had recieved lenalidomide therapy with 75% erythroid response and 21% complete cytogenetic response [7]; peripheral blood or bone marrow samples from these patients were retrospectively screened for *TP53* mutations with 6 (25%) patients expressing the mutation at diagnosis and another 9 (38%) during follow-up, of whom, one also manifested a new *RUNX1* mutation, as was the case in the current report. The particular study showed appearance of *TP53* mutations in 9 (50%) of 18 patients in whom the mutation was not detected at diagnosis and the authors were able to show correlation between *TP53* clonal evolution and disease progression [7]. Interestingly, in a more recent report of 416 patients with therapy-related myeloid neoplasms, the authors described an association between *TP53* mutations and prior treatment with thalidomide analogs, specifically lenalidomide, and the authors were able to provide experimental evidence for lenalidomide-induced selective advantage for *TP53* mutant clones [6]. Taken together, these observations suggest lenalidomide-enhanced expansion of pre-existing mutant *TP53* clones that might be less sensitive to lenalidomide-induced clonal suppression in MDS-del(5q). However, none of the aforementioned studies were controlled and do not necessarily prove cause and effect and, instead, the particular phenomenon might represent clonal evolution as an intrinsic disease biology that may or may not be influenced by specific therapy. The latter impression is consistent with the observations from the current study, which suggest lenalidomide treatment-emergent mutations might not be restricted to *TP53* but also involve other genes.

Current practice in the real-world setting, in regard to patients with MDS-del(5q), is heterogeneous, in terms of both treatment initiation with lenalidomide and disease monitoring [12, 13]. The observations from the current study underline the need for monitoring of mutations both at diagnosis (preferably using highly sensitive assays) and during the disease course, in patients with MDS-del(5q), regardless of specific treatment. Actionable events include the presence of *TP53* VAF ≥ 22%, which requires intervention with allogeneic stem cell transplant [14], and utilization of alternative therapy, such as hypomethylating agents [6], which might be active in the absence of complex karyotype [15].

## Data Availability

Data might be requested by email to the corresponding author.
